# Association between the CRP–TyG index and lupus nephritis risk

**DOI:** 10.17305/bb.2025.13043

**Published:** 2025-10-20

**Authors:** Baozhu Liang, Ming Tang, Junqi Huang, Yingfei Li, Rongmei Liang, Zeqing Zhai, Erwei Sun

**Affiliations:** 1 Department of Rheumatology and Immunology, The Third Affiliated Hospital, Southern Medical University, Guangzhou, Guangdong, China; 2 Guangdong Provincial Key Laboratory of Bone and Joint Degeneration Diseases, The Third Affiliated Hospital, Southern Medical University, Guangzhou, Guangdong, China

**Keywords:** C-reactive protein–triglyceride–glucose index, systemic lupus erythematosus, lupus nephritis, proteinuria.

## Abstract

Assessment of insulin resistance is increasingly emphasized in patients with systemic lupus erythematosus (SLE) due to its significant role in predicting kidney injury and cardiovascular risk. Given that sustained inflammation is a hallmark of SLE, the novel C-reactive protein (CRP)–triglyceride–glucose (TyG) index (CTI), which comprehensively reflects insulin resistance and inflammation, has emerged as a valuable biomarker. This study aimed to investigate the association between the CTI and the risk of lupus nephritis (LN) risk and further explore its predictive potential in SLE patients. A cohort of 195 SLE patients stratified by renal involvement or CTI tertiles were included. Spearman’s correlation analysis was performed to assess the relationship between the CTI and clinical parameters of lupus activity. Logistic regression analysis was utilized to identify the association between the CTI and risk of LN. The receiver operating characteristic (ROC) curve was employed to evaluate the CTI and the TyG index in predicting LN. The results demonstrated significantly elevated CTI levels in the LN group compared to the non-LN group. Multivariate-adjusted regression analysis indicated that a unit increase in CTI corresponded to enhanced risk of LN (adjusted odds ratio (OR) ═ 2.062; 95% confidence interval (CI): 1.208–3.522), particularly among patients in the third tertile compared to those in the first tertile (adjusted OR = 4.368; 95% CI: 1.411–13.520). Subgroup analysis revealed that SLE patients with a SLEDAI-2K score greater than 6 exhibited an increased LN risk associated with higher CTI levels. ROC analysis illustrated the higher sensitivity of CTI (area under the curve [AUC] ═ 0.6592; 95% CI: 0.576–0.742) compared to the TyG index (AUC = 0.6327; 95% CI: 0.546–0.719) in predicting LN risk. These findings indicate that elevated CTI is strongly associated with an increased risk of LN, suggesting its potential as a valuable predictor of LN risk in SLE patients.

## Introduction

Systemic lupus erythematosus (SLE) is an autoimmune disease characterized by the production of multiple autoantibodies, significant organ damage, frequent disease flares, and persistent inflammation [1, 2]. Lupus nephritis (LN) is one of the most common and severe manifestations in patients with SLE, potentially leading to end-stage renal disease and a marked increase in mortality risk [3, 4]. Therefore, early diagnosis and effective treatment strategies for kidney involvement are critical for improving the prognosis of these patients.

Metabolic disturbances, including insulin resistance and dyslipidemia, have garnered increased attention in SLE patients due to their positive correlation with disease progression. Insulin resistance contributes to complement activation, immune dysregulation, and the enhanced secretion of inflammatory cytokines, which may exacerbate systemic inflammation and nephritis activity [5–9].

The triglyceride–glucose (TyG) index is a clinically valuable marker for assessing insulin resistance. Emerging evidence indicates that an elevated TyG index is associated with renal impairment, including higher proteinuria levels and accelerated chronic kidney disease (CKD) progression in diabetic populations [10–12]. Specifically, the TyG index correlates with an increased risk of carotid atherosclerosis, retinal microvascular damage, and hypertension in patients with autoimmune diseases [13–15]. Recently, the C-reactive protein–TyG index (CTI), which integrates insulin resistance and systemic inflammation, has been identified as a valuable predictor of cardiovascular disease, stroke risk, and liver fibrosis progression [16–18]. The CTI reflects systemic inflammation and metabolic dysfunction by incorporating C-reactive protein (CRP) and TyG index parameters. However, evidence regarding the association between CTI and LN risk in SLE patients is limited. This study aims to investigate the association and further explore the predictive efficacy of CTI concerning LN risk in SLE patients.

## Materials and methods

### Study population

This retrospective study enrolled 195 patients with SLE from the Third Affiliated Hospital of Southern Medical University between January 2018 and December 2022. Inclusion criteria were: (1) fulfillment of the European League Against Rheumatism/American College of Rheumatology (EULAR/ACR) classification criteria (2019) for SLE; (2) availability of data for fasting triglycerides, glucose concentrations, and CRP levels; and (3) age over 18 years. Patients classified in the LN group were required to meet additional criteria: (1) persistent proteinuria exceeding 0.5 g/day or urine protein >3+ in routine urinalysis; and/or persistent cellular casts; or active urinary sediment (defined as >5 red blood cells/high-power field, >5 white blood cells/high-power field, with infection excluded); and/or renal biopsy pathology confirming LN. Exclusion criteria included: (1) pregnancy; (2) age under 18; (3) history of gastrointestinal surgery; (4) severe liver dysfunction; (5) history of malignancy; (6) history of diabetes; (7) history of hypertension; and (8) current infections. The SLE disease activity index-2000 (SLEDAI-2K) score was utilized to evaluate disease activity among SLE patients.

### Data collection

Demographic and clinical characteristics of SLE patients, along with laboratory data, were collected from medical records. Collected data included age, sex, CRP levels, erythrocyte sedimentation rate (ESR), anti-dsDNA antibody, anti-Sm antibody, 24-h proteinuria, blood creatinine, blood urea nitrogen, blood uric acid, estimated glomerular filtration rate (eGFR), low-density lipoprotein cholesterol (LDL-C), high-density lipoprotein cholesterol (HDL-C), fasting triglycerides (TG), total cholesterol (TC), fasting glucose, complements (C3, C4, and C1q), and 25-hydroxyvitamin D (25(OH)D).

### TyG index and CTI assessment

The TyG index was calculated using the following formula: Ln [Blood triglyceride concentration (mg/dL) × Blood glucose concentration (mg/dL)/2]. The CTI was calculated according to the following formula: TyG index + 0.412 × Ln [CRP (mg/L)] [17].

### eGFR assessment

The eGFR was assessed using the CKD Epidemiology Collaboration (CKD-EPI) equation (2021) [19].

### Ethical statement

This study was conducted in accordance with the ethical principles outlined in the *Declaration of Helsinki* and relevant national clinical research regulations. Ethical approval was granted by the Ethics Committee of the Third Affiliated Hospital of Southern Medical University.

### Statistical analysis

Statistical analyses were performed using the Statistical Package for the Social Sciences (SPSS) software version 23.0. Graphs were produced using GraphPad Prism software version 8 and RStudio. The extent of missing data in this study is presented in Table S1, and complete cases were utilized to address missing values. Continuous variables were expressed as mean ± standard deviation (SD) or median (interquartile range, IQR), while categorical variables were presented as counts or percentages. Normality of data was assessed using the Shapiro–Wilk test. Comparisons between the LN and non-LN groups were conducted using Student’s *t*-test for symmetrically distributed data and the Mann–Whitney *U* test for skewed data. Comparisons across CTI tertiles employed ANOVA for continuous parametric variables and the Kruskal–Wallis test for nonparametric data. Categorical variables were compared using the chi-square test. Correlations between CTI and clinical parameters were evaluated using Spearman’s correlation coefficient. To identify risk factors associated with LN, logistic regression models were utilized, with results expressed as odds ratios (ORs) and 95% confidence intervals (CIs). Based on the logistic model with continuous CTI values and 65 observed LN events, power calculations were performed assuming a two-tailed α of 0.05, yielding a post-hoc power of 91.5%, confirming sufficient coefficient stability for analysis. Three logistic models were employed to explore the association between CTI and LN: Model 1 was unadjusted, Model 2 adjusted for age and sex, and Model 3 adjusted for age, ESR, anti-dsDNA antibody, glucocorticoid use, and lipid-lowering agents. Variance inflation factors (VIFs) were used to assess potential collinearity between CTI and other covariates, with all covariates demonstrating VIFs less than 5 (as shown in Table S2), indicating no significant multicollinearity in regression models. Corresponding ORs and 95% CIs are presented in Table S3. The Hosmer–Lemeshow test was employed to evaluate the calibration of logistic regression models incorporating CTI for discriminative performance in identifying LN, with results indicating adequate calibration of prediction models (all *P* > 0.05, as shown in Table S4). To evaluate potential effect modification, stratified analyses were performed for covariates (age, eGFR, SLEDAI-2K scores, and glucocorticoid use) on the relationship between CTI and LN risk. The distributions of LN and non-LN events in each subgroup are summarized in Table S5. The Benjamini–Hochberg false-discovery rate was applied to control for multiplicity, including CTI tertile comparisons, correlation analyses, and subgroup interaction tests. Additionally, receiver operating characteristic (ROC) analysis was performed to assess the predictive capacity for LN risk, with the area under the ROC curve (AUC) evaluating the incremental effect of CTI. The restricted cubic spline (RCS) with four knots (at the 5th, 35th, 65th, and 95th percentiles) was employed to explore the dose–response relationship between CTI and LN risk. The RCS plot was created in RStudio using the rms R package. A *P* value <0.05 was considered statistically significant.

**Table 1:** Baseline characteristics of the SLE patients with or without LN

**Parameter**	**LN** **(*n* ═ 65)**	**Non-LN** **(*n* ═ 130)**	***P* value**
*Demographic characteristics*			
Age (years)	31.60 ± 9.33	34.26 ± 10.59	0.087
Sex			0.504
Male, *n* (%)	8 (12.3)	12 (9.2)	
Female, *n* (%)	57 (87.7)	118 (90.8)	
*Clinical features*			
SLEDAI-2K score	18 (8.5–22)	6.5 (3.25–10)	**<0.001**
Glucocorticoid treatment, *n* (%)	52 (80.0)	86 (66.2)	**0.045**
*Renal function*			
eGFR (mL/min/1.73 m^2^)	96.4 (68.78–120.08)	117.15 (96.95–125.5)	**<0.001**
24-h proteinuria (mg/d)	736.81 (201.18–1840.31)	109.64 (78.90–155.12)	**<0.001**
BUN (mmol/L)	6.02 (4.46–9.62)	4.14 (3.51–5.25)	**<0.001**
Cr (µmol/L)	75 (63.5–88.5)	58.5 (51–73)	**<0.001**
UA (µmol/L)	406.5 (315.75–478.75)	340.5 (276–386.25)	**<0.001**
Hematuria, *n* (%)	48 (73.8)	40 (30.8)	**<0.001**
*Inflammation and immunity*			
ESR (mm/h)	39 (18.25–71.5)	21 (12.25–29.75)	**0.001**
CRP (mg/L)	2.05 (0.54–7.91)	0.99 (0.39–3.00)	**0.009**
C3 (g/L)	0.53 (0.37–0.97)	0.86 (0.64–0.97)	**<0.001**
C4 (g/L)	0.09 (0.04–0.21)	0.14 (0.1–0.24)	**0.003**
C1q (mg/L)	145.54 ± 33.85	158.67 ± 36.44	**0.016**
Anti-dsDNA antibody (IU/mL)	36.86 (1.82–283.21)	9.46 (2.08–41.56)	**0.001**
Anti-Sm antibody (RU/mL)	2.64 (2–23.47)	3.49 (2–51.80)	0.568
D-dimer (µg/L)	329.5 (135–764.5)	168 (112–348.5)	**<0.001**
*Metabolic profile*			
GLU (mg/dL)	78.56 (72.07–89.01)	81.98 (74.41–87.21)	0.542
TG (mg/dL)	123.11 (80.60–178.91)	90.34 (64.66–124.00)	**0.001**
TyG index	8.51 ± 0.61	8.22 ± 0.49	**0.002**
CTI	8.78 ± 0.97	8.25 ± 0.88	**<0.001**
TC (mmol/L)	4.38 (3.83–5.08)	3.82 (3.18–4.46)	**<0.001**
LDL-C (mmol/L)	2.46 (2.02–3.16)	2.2 (1.47–2.87)	**0.001**
HDL-C (mmol/L)	1.1 (0.87–1.49)	1.09 (0.91–1.30)	0.539
25(OH)D (nmol/L)	48.05 ± 20.01	55.72 ± 17.69	**0.007**

Data were presented as *n*, median (interquartile range, IQR) or mean ± standard deviation. Abbreviations: LN: Lupus nephritis; SLEDAI-2K: Systemic lupus erythematosus disease activity index-2000; eGFR: Estimated glomerular filtration rate; BUN: Blood urea nitrogen; Cr: Creatinine; UA: Uric acid; ESR: Erythrocyte sedimentation rate; CRP: C-reactive protein; C3: Complement 3; C4: Complement 4; GLU: Glucose; TG: Triglyceride; TyG index: Triglyceride–glucose index; CTI: CRP–triglyceride–glucose index; TC: Total cholesterol; LDL-C: Low-density lipoprotein cholesterol; HDL-C: High-density lipoprotein cholesterol; 25(OH)D: 25-HydroxyvitaminD; SLE: Systemic lupus erythematosus.

## Results

### Demographic and clinical characteristics of SLE patients according to LN status and CTI tertiles

We analyzed a cohort of 195 SLE patients with available CTI data. As presented in Table 1, patients in the LN group exhibited significantly higher levels of SLEDAI-2K scores, 24-h proteinuria, BUN, Cr, UA, ESR, CRP, anti-dsDNA antibody, D-dimer, TG, TC, and LDL-C compared to those in the non-LN group (all *P* < 0.01). Conversely, LN patients had significantly lower levels of eGFR, C3, C4, C1q, and 25(OH)D (all *P* < 0.05). Overall, SLE patients with LN had a significantly higher CTI than those without (8.78 ± 0.97 vs 8.25 ± 0.88, *P* < 0.001). We further categorized the patients into three groups based on CTI tertiles, with average CTI values of 6.31–7.88 in tertile 1, 7.88–8.93 in tertile 2, and 8.93–10.98 in tertile 3. Table 2 summarizes the comparisons of demographic, laboratory, and clinical characteristics among the CTI tertiles. Notably, we observed significantly elevated levels of SLEDAI-2K scores, UA, ESR, CRP, anti-dsDNA antibody, GLU, TG, and TC, alongside reduced levels of eGFR and HDL-C as CTI tertiles increased (all *P* < 0.05).

**Table 2:** Clinical and laboratory characteristics based on CTI tertiles

**Parameter**	**Overall** **(*n* ═ 195)** **(6.31–10.98)**	**CTI**			***P* value**
		**Tertile 1** **(*n* ═ 65)** **(6.31–7.88)**	**Tertile 2** **(*n* ═ 65)** **(7.88–8.93)**	**Tertile 3** **(*n* ═ 65)** **(8.93–10.98)**	
*Demographic characteristics*					
Age (years)	33.37 ± 10.24	31.6 ± 9.31	34.14 ± 12.09	34.38 ± 8.97	0.085
Sex					0.946
Male, *n* (%)	20 (10.3)	7 (10.8)	7 (10.8)	6 (9.2)	
Female, *n* (%)	175 (89.7)	58 (89.2)	58 (89.2)	59 (90.8)	
*Clinical features*					
SLEDAI-2K score	9 (4–16)	5 (2–10)	8 (4–14.5)^†^	14 (7.25–20)^† †, #^	**<0.001**
*Renal function*					
BUN (mmol/L)	4.76 (3.72–6.49)	4.61 (3.38–6.04)	4.38 (3.73–5.37)	5.63 (4.13–7.28)^†^	**0.021**
Cr (µmol/L)	65 (52.02–79.75)	59 (51–68)	60.5 (50.5–78.25)	75 (60.75–100.25)	0.081
UA (µmol/L)	345.5 (296.25–439.75)	308 (269–416)	344 (315–439.25)	367 (311.5–474.75)^†^	**0.025**
eGFR (mL/min/1.73 m^2^)	111.95 (84.18–123.25)	117.3 (100.7–125.5)	117.55 (89.5–123.25)	87.05 (62.85–116.1)^†^	**0.025**
24-h proteinuria (mg/d)	152.65 (88.36–495.53)	122.15 (69.12–176.39)	116.105 (82.92–427.52)	284.305 (154.30–1062.78)^† †, ##^	**<0.001**
*Inflammation and immunity*					
ESR (mm/h)	25 (14–42.75)	18 (12–25)	20.5 (10–27.25)	45.5 (29.25–71.5)^†, #^	**<0.001**
CRP (mg/L)	1.25 (0.43–4.00)	0.28 (0.19–0.53)	1.36 (0.58–2.19)^† † †^	7 (3.23–12.19)^† † †, ###^	**<0.001**
C3 (g/L)	0.81 (0.51–0.97)	0.78 (0.57–0.97)	0.9 (0.50–1.08)	0.74 (0.47–0.93)	0.256
C4 (g/L)	0.13 (0.07–0.23)	0.15 (0.1–0.24)	0.12 (0.07–0.21)	0.12 (0.06–0.20)	0.085
C1q (mg/L)	142 (124.25–164.5)	145 (128–166)	139 (115.75–164.5)	141.5 (126.5–163.75)	0.797
Anti-dsDNA antibody (IU/mL)	13.95 (2.01–93.81)	4.17 (1–47.67)	15.5 (1.75–84.65)	28.01 (2.59–264.78)^†^	**0.002**
Anti-Sm antibody (RU/mL)	3.18 (2–28.51)	5.62 (2–59.35)	2 (2–10.36)	3.35 (2–35.94)	0.435
D-dimer (µg/L)	182 (124–477.75)	159 (106–329.5)	147 (100–335)	429.5 (156–1213.5)^† †, ##^	**<0.001**
*Metabolic profile*					
GLU (mg/dL)	80.90 (72.97–87.39)	77.66 (71.17–84.50)	80.36 (71.89–86.58)	85.23 (79.01–95.14)^† †, ##^	**<0.001**
TG (mg/dL)	95.66 (70.86–142.60)	65.54 (55.36–85.47)	103.63 (76.17–124.00)^† †^	148.80 (112.04–211.24)^† †, #^^#^	**<0.001**
TC (mmol/L)	4.03 (3.41–4.78)	3.91 (3.32–4.59)	4.15 (3.54–4.62)	4.34 (3.47–5.20)^†^	**0.032**
LDL-C (mmol/L)	2.35 (1.78–3.01)	2.15 (1.63–2.75)	2.20 (1.99–2.92)	2.41 (1.64–3.12)	0.736
HDL-C (mmol/L)	1.09 (0.91–1.32)	1.28 (0.97–1.46)	1.11 (0.93–1.33)	1.02 (0.83–1.31)	**0.032**
25(OH)D (nmol/L)	53.16 ± 18.80	55.58 ± 18.61	49.85 ± 19.65	54.05 ± 17.91	0.632
TyG index	8.32 ± 0.55	7.88 ± 0.30	8.29 ± 0.45 ^† †^	8.79 ± 0.45 ^† † †, ###^	**<0.001**
CTI	8.43 ± 0.94	7.40 ± 0.40	8.38 ± 0.29 ^† †^	9.51 ± 0.42 ^† † †, ###^	**<0.001**

Data were presented as *n*, median (interquartile range, IQR) or mean ± standard deviation. ^†^, compared with tertile 1, *P* < 0.05; ^† †^, *P* < 0.01; ^† † †^, *P* < 0.001. ^#^, compared with tertile 2, ^##^, *P* < 0.01; ^###^, *P* < 0.001. Abbreviations: SLEDAI-2K: Systemic lupus erythematosus disease activity index-2000; eGFR: Estimated glomerular filtration rate; BUN: Blood urea nitrogen; Cr: Creatinine; UA: Uric acid; ESR: Erythrocyte sedimentation rate; CRP: C-reactive protein; C3: Complement 3; C4: Complement 4; GLU: Glucose; TG: Triglyceride; CTI: CRP–triglyceride–glucose index; TyG index: Triglyceride–glucose index; TC: Total cholesterol; LDL-C: Low-density lipoprotein cholesterol; HDL-C: High-density lipoprotein cholesterol; 25(OH)D: 25-HydroxyvitaminD.

### Correlations of CTI with clinical parameters in SLE patients

**Figure 1.**
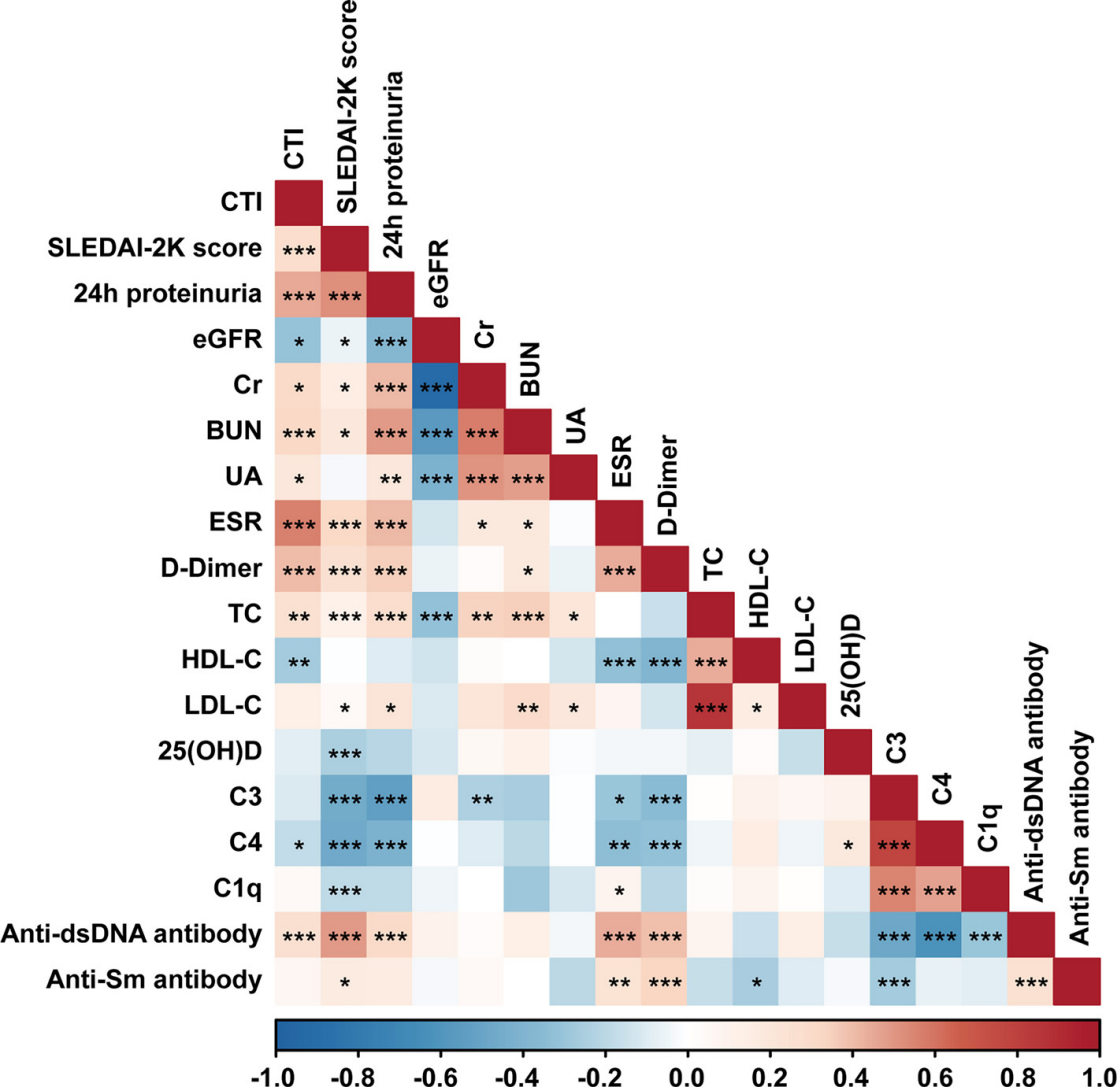
**Correlations between the CTI and clinical parameters in SLE patients.** *P* values were adjusted for the Benjamini–Hochberg false discovery rate (FDR) method. *P < 0.05, **P < 0.01, ***P < 0.001. Abbreviations: CTI: C-reactive protein (CRP)–triglyceride–glucose index; SLEDAI-2K: Systemic lupus erythematosus disease activity index-2000; eGFR: Estimated glomerular filtration rate; BUN: Blood urea nitrogen; Cr: Creatinine; UA: Uric acid; ESR: Erythrocyte sedimentation rate; C3: Complement 3; C4: Complement 4; TC: Total cholesterol; LDL-C: Low-density lipoprotein cholesterol; HDL-C: High-density lipoprotein cholesterol; 25(OH)D: 25-HydroxyvitaminD; SLE: Systemic lupus erythematosus.

Figure 1 illustrates Spearman’s correlations between CTI and clinical parameters in SLE patients. CTI demonstrated positive correlations with SLEDAI-2K scores, 24-h proteinuria, Cr, BUN, UA, ESR, D-Dimer, TC, and anti-dsDNA antibody levels, while exhibiting negative correlations with eGFR, HDL-C, and C4 levels (all *P* < 0.05).

**Figure 2.**
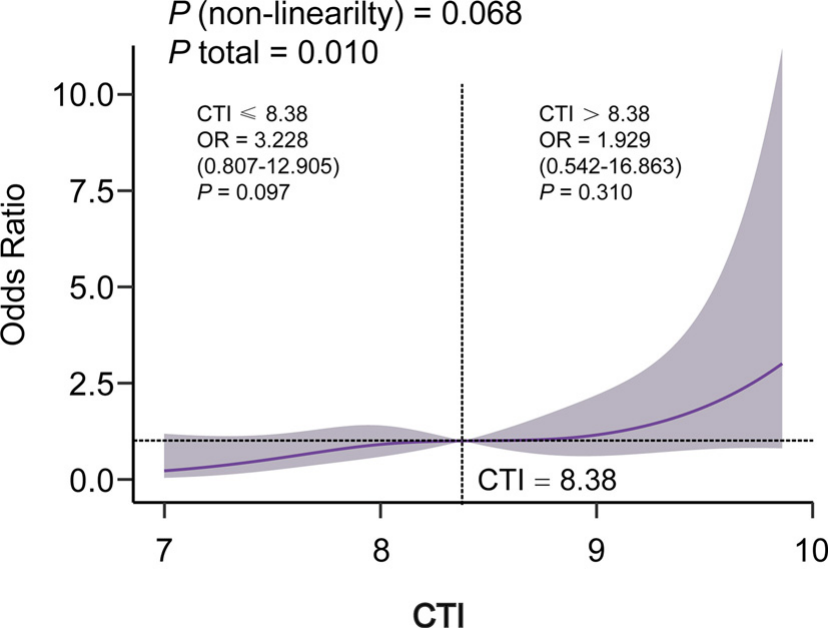
**Restricted cubic spline curves for the CTI and LN.** Adjusted for age, erythrocyte sedimentation rate, anti-dsDNA antibody, use of glucocorticoid, and use of lipid-lowering agents. Abbreviations: CTI: C-reactive protein–triglyceride–glucose index; OR: Odds ratio; LN: Lupus nephritis.

### Relationship between CTI and LN risk in SLE patients

Table 3 presents the multivariate regression analysis examining the relationship between CTI and LN risk. After adjusting for all covariates in Model 3, each unit increase in CTI was associated with a 106.2% increase in LN risk (OR = 2.062, 95% CI: 1.208–3.522), and each SD increase in CTI (SD = 0.94) predicted a 97.5% increase in LN risk (OR = 1.975, 95% CI: 1.194–3.266). We subsequently stratified CTI into tertiles for sensitivity analysis. Compared to tertile 1, tertile 3 demonstrated a significant association with increased LN risk (OR = 3.879; 95% CI: 1.781–8.447, *P* for trend 0.002) in Model 1, and after adjusting for age and sex in Model 2 (OR = 4.497; 95% CI: 2.016–10.03, *P* for trend 0.001). Furthermore, this positive association remained significant after adjusting for all covariates in Model 3 (OR = 4.368; 95% CI: 1.411–13.520, *P* for trend 0.031). Additionally, the RCS regression model was employed to assess the association between CTI and LN risk (shown in Figure 2), revealing a dose–response relationship in the fully adjusted Model 3 (*P* for total = 0.010, *P* for nonlinearity = 0.068).

**Table 3:** Odds ratios for the association of the CTI with LN in patients with SLE

	**Per 1 unit CTI increase**	**Per 1 SD CTI increase**	**CTI**			***P* for trend**
			**Tertile 1 (*n* ═ 65) (6.31–7.88)**	**Tertile 2 (*n* ═ 65) (7.88–8.93)**	**Tertile 3 (*n* ═ 65) (8.93–10.98)**	
*OR* (95% *CI*), *P value*						
Model 1	1.879 (1.331–2.653) *P* < 0.001	1.810 (1.309–2.502) *P* < 0.001	Ref.	1.778 (0.795–3.973) *P* ═ 0.161	3.879 (1.781–8.447) *P* ═ 0.001	0.002
Model 2	2.004 (1.407–2.853) *P* < 0.001	1.922 (1.379–2.680) *P* < 0.001	Ref.	1.938 (0.856–4.388) *P* ═ 0.113	4.497 (2.016–10.03) *P* ═ 0.001	0.001
Model 3	2.062 (1.208–3.522) *P* ═ 0.008	1.975 (1.194–3.266) *P* ═ 0.008	Ref.	2.857 (1.034–7.893) *P* ═ 0.043	4.368 (1.411–13.520) *P* ═ 0.011	0.031

Model 1: No covariates were adjusted; Model 2: Adjusted for age and sex; Model 3: Adjusted for age, erythrocyte sedimentation rate (ESR), anti-dsDNA antibody, use of glucocorticoid, and use of lipid-lowering agents. Abbreviations: LN: Lupus nephritis; SLE: Systemic lupus erythematosus; CTI: C-reactive protein–triglyceride–glucose index; CI: Confidence interval; OR: Odds ratio.

**Table 4:** Associations between the CTI and LN in subgroup analysis

**Variables**	***N***	**OR (95% CI)**	***P* value**	***P* for interaction**
Age (years)				0.813
>35	70	4.357 (1.906–9.958)	0.001	
≤35	125	1.608 (1.054–2.453)	0.028	
eGFR (mL/min/1.73 m^2^)				0.005
eGFR < 60	21	1.351 (0.545–3.348)	0.516	
60 ≤ eGFR < 90	30	1.340 (0.593–3.028)	0.482	
eGFR ≥ 90	144	1.901 (1.213–2.978)	0.005	
SLEDAI-2K score				<0.001
>6	102	1.535 (1.017–2.315)	0.041	
≤6	93	1.827 (0.906–3.686)	0.092	
Use of glucocorticoid				0.089
Yes	138	2.480 (1.146–5.369)	0.021	
No	57	1.672 (1.135–2.464)	0.009	

Abbreviations: eGFR: Estimated glomerular filtration rate; SLEDAI-2K: Systemic lupus erythematosus disease activity index-2000; LN: Lupus nephritis; CTI: C-reactive protein–triglyceride–glucose index; CI: Confidence interval; OR: Odds ratio.

### Subgroup analysis

Table 4 details the subgroup analyses evaluating the stability of the association between CTI and LN risk in SLE patients. Patients were categorized into subgroups based on age, eGFR, disease activity (SLEDAI-2K scores), and glucocorticoid use. When stratified by age or glucocorticoid use, interaction *P* values were non-significant (both *P* > 0.05), indicating no modification of the association by these factors. In eGFR subgroups, patients with preserved renal function (eGFR ≥ 90 mL/min/1.73 m^2^) exhibited a significantly higher LN risk associated with elevated CTI (OR = 1.901, 95% CI: 1.213–2.978, *P* ═ 0.005). Importantly, disease activity significantly interacted with this association (*P* for interaction < 0.001), where patients with high disease activity (SLEDAI-2K score > 6) showed significantly elevated LN risk (OR = 1.535, 95% CI: 1.017–2.315, *P* ═ 0.041) compared to those with low disease activity (SLEDAI-2K score ≤ 6). These findings suggest that patients with active SLE represent a high-risk subgroup for CTI-associated LN development.

### Potential predictive value of CTI for LN risk

**Figure 3.**
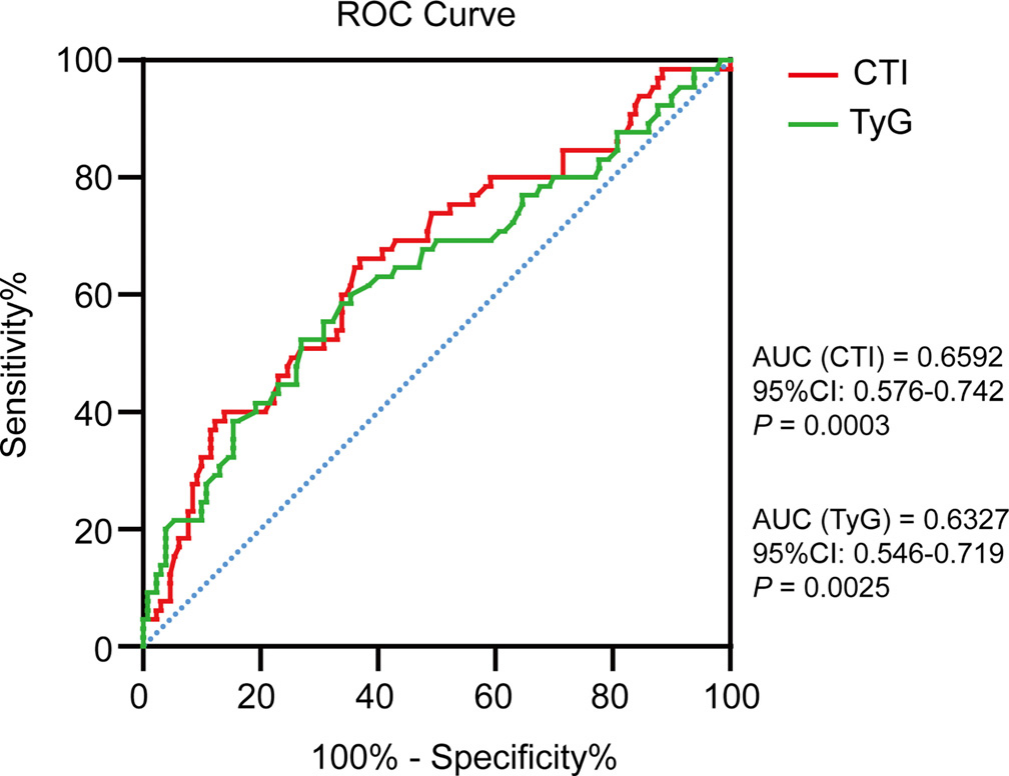
**The ROC curves of the CTI and TyG index for predicting LN in patients with SLE.** ROC analysis comparing CTI (red) and TyG (green). CTI showed an AUC of 0.6592 (95% CI 0.576–0.742), while TyG had an AUC of 0.6327 (95% CI 0.546–0.719). Optimal cut-offs: CTI 8.46 (sensitivity 66.2%, specificity 63.1%) vs TyG 8.50 (sensitivity 52.3%, specificity 73.1%). Abbreviations: TyG: Triglyceride–glucose; AUC: Area under the curve; CI: Confidence interval; LN: Lupus nephritis; SLE: Systemic lupus erythematosus; ROC: Receiver operating characteristic; CTI: C-reactive protein–triglyceride–glucose index.

ROC curves were generated to directly compare the predictive capabilities of CTI and the TyG index for LN risk. As depicted in Figure 3, the CTI exhibited an area under the curve (AUC) of 0.6592 (95% CI: 0.576–0.742), while the TyG index demonstrated an AUC of 0.6327 (95% CI: 0.546–0.719). The CTI cut-off value of 8.46 achieved a sensitivity of 66.2% and specificity of 63.1%, and the TyG index cut-off of 8.50 (sensitivity = 52.3%, specificity = 73.1%). CTI exhibited better sensitivity for detecting LN risk compared to the TyG index, which may significantly enhance early identification of high-risk patients.

## Discussion

Kidney involvement is among the most common and severe clinical manifestations of SLE, posing significant challenges to achieving disease remission. End-stage renal disease remains a leading cause of mortality in this population [20, 21]. While invasive renal biopsy is the diagnostic gold standard for LN, the risk of procedure-related complications necessitates careful consideration. These limitations underscore the urgent need for non-invasive and accurate predictive markers to facilitate earlier diagnosis of LN.

Previous studies have identified the TyG index as a crucial metabolic marker for predicting renal damage in populations with diabetes and cardiovascular disease [10, 22–24]. Recently, the TyG index has been validated as a reliable screening tool for insulin resistance in patients with SLE [25], demonstrating its predictive value for SLE comorbidities such as cardiovascular risk, myosteatosis, hypertension incidence, and LN risk [9, 14, 15]. Specifically, CTI synergistically integrates CRP and the TyG index parameters, thus comprehensively reflecting systemic inflammation and metabolic dysfunction. Chronic inflammation constitutes a hallmark of SLE pathogenesis, especially in LN progression, but the association between CTI and LN risk remains unexplored.

In our study, we observed significantly elevated CTI in LN patients compared to non-LN patients. Also, CTI demonstrated strong positive correlations with LDL-C, TC, ESR and anti-dsDNA antibody, alongside a negative correlation with C3 and HDL-C levels in SLE patients. These findings align with prior research indicating that insulin resistance and activated inflammation play critical roles in the pathogenesis of LN [26–28]. Moreover, our data suggest that CTI may serve as a predictive indicator for identifying LN, exhibiting higher sensitivity compared to the TyG index. Subgroup analysis revealed a robust association between CTI and LN, particularly in patients with high disease activity (SLEDAI-2K score > 6) or preserved renal function (eGFR ≥ 90 mL/min/1.73 m^2^), underscoring the relevance of CTI in autoimmune-driven renal injury, especially among active SLE patients.

Emerging evidence indicates that insulin resistance exacerbates renal injury through both non-immunological and immunomodulatory pathways. Initially, insulin resistance can induce non-immune renal damage via adipocytokine-driven fibrotic remodeling, mitochondrial dysfunction-induced oxidative stress, accumulation of advanced glycation end-products, and vitamin D deficiency [8, 26, 28–31]. Our research demonstrated that LN patients exhibited metabolic dysregulation, characterized by elevated TC, TG and LDL-C, alongside decreased 25(OH)D levels compared to non-LN patients, which may mechanistically contribute to renal injury independent of autoimmunity. Furthermore, insulin resistance can disrupt immune homeostasis through enhanced immune-complex deposition, complement activation, and inflammation amplification [32, 33]. Specifically, a self-perpetuating cycle can form between inflammation and insulin resistance, where sustained inflammatory cytokines worsen insulin resistance, and hyperglycemia along with hyperinsulinism amplify inflammatory responses. Collectively, these pathways contribute to renal hemodynamic dysregulation, glomerular endothelial damage, and podocyte foot process fusion, driving LN progression.

Although insulin resistance is implicated in LN, therapeutic strategies directly targeting insulin resistance for LN management remain limited. Among conventional immunomodulators used in LN treatment, hydroxychloroquine (HCQ) has been shown to ameliorate insulin resistance, whereas glucocorticoids exert an opposing effect [34, 35]. This highlights the need to address glucocorticoid-induced metabolic complications and the clinical significance of exploring novel therapies specifically targeting insulin resistance in SLE patients.

Despite a comprehensive analysis of the association between CTI and LN risk in this study, several limitations warrant mention. First, as a component of CTI, CRP reflects SLE disease activity and nonspecific inflammation [36]. CRP levels are primarily driven by interleukin-6 (IL-6) in active SLE, and significantly elevated CRP levels are associated with renal damage in SLE [37]. However, the production of CRP is influenced by various regulatory factors, including interferon-dependent suppression of hepatic CRP production and concurrent infections [36, 38], which may limit its significance as a reliable inflammation marker in SLE patients. Second, the limited availability of renal biopsy classifications among patients in our study restricted the examination of correlations between CTI and renal histopathological outcomes. Additionally, this retrospective cross-sectional study included only Chinese individuals and was conducted at a single research center. The limited sample size also restricted statistical power, particularly for subgroup analyses. Future multicenter studies with larger, ethnically diverse cohorts are necessary to validate these findings. Finally, our study primarily focused on the relationship between CTI and LN risk while lacking longitudinal data to establish causation.

## Conclusion

In conclusion, this study identified CTI as a biomarker integrating inflammation and metabolic dysfunction that can independently predict the risk of LN. Our findings demonstrate a significant association between elevated CTI levels and increased LN risk. CTI may provide valuable insights for identifying SLE patients at heightened risk of renal involvement and serve as an effective and straightforward indicator for LN risk assessment in clinical practice. Future research is warranted to explore the causal and mechanistic links between CTI and renal immune disorders.

## Supplemental data

**Table S1:** Distribution of variables with missing data

**Variables**	**Number of missing**	**Missing proportion**
24-h proteinuria	33	16.9%
BUN	28	14.4%
UA	1	0.5%
ESR	3	1.5%
C3	1	0.5%
C4	1	0.5%
C1q	2	1.0%
D-dimer	15	7.7%
Anti-Sm antibody	43	22.1%

Abbreviations: BUN: Blood urea nitrogen; Cr: Creatinine; UA: Uric acid; ESR: Erythrocyte sedimentation rate; CRP: C-reactive protein; C3: Complement 3; C4: Complement 4.

**Table S2:** The VIF values for all variables

**Covariates**	**β**	***P* value**	**VIF**
Sex	0.107	0.171	1.035
Age	0.020	0.795	1.049
ESR	0.316	<0.001	1.112
Anti-dsDNA antibody	0.092	0.247	1.071
Use of glucocorticoid	0.056	0.469	1.016
Use of lipid-lowering agents	0.097	0.210	1.028

Abbreviations: VIF: Variance inflation factor; ESR: Erythrocyte sedimentation rate.

**Table S3:** The ORs and 95% CIs for all variables in Model 3

**Variables**	**OR (95% CI)**	***P* value**
Age	0.975 (0.933–1.018)	0.251
ESR	1.010 (0.992–1.029)	0.286
Anti-dsDNA antibody	1.000 (1.000–1.001)	0.359
Use of glucocorticoid	1.244 (0.516–2.999)	0.627
Use of lipid-lowering agents	0.000	0.999

Abbreviations: ORs: Odds ratios; CIs: Confidence intervals; ESR: Erythrocyte sedimentation rate.

**Table S4:** Hosmer–Lemeshow test of the models

**Models**	**χ^2^**	***P* value**
Model 1	9.196	0.326
Model 2	7.778	0.353
Model 2 + CTI	4.430	0.816
Model 3	14.691	0.065
Model 3 + CTI	10.215	0.250

Model 1: No covariates were adjusted; Model 2: Adjusted for age and sex; Model 3: Adjusted for age, erythrocyte sedimentation rate, anti-dsDNA antibody, use of glucocorticoid, and use of lipid-lowering agents. Abbreviation: CTI: C-reactive protein–triglyceride–glucose index.

**Table S5:** The distributions of LN and non-LN events in each subgroup

**Variables**	**LN events**	**Non-LN events**
*Age (years)*		
>35	16	54
≤35	49	76
*eGFR (mL/min/1.73 m^2^)*		
eGFR < 60	14	7
60 ≤ eGFR < 90	15	15
eGFR < 60	36	108
*SLEDAI-2K score*		
>6	50	52
≤6	15	78
*Use of glucocorticoid*		
Yes	52	86
No	13	44

Abbreviations: LN: Lupus nephritis; eGFR: Estimated glomerular filtration rate; SLEDAI-2K: Systemic lupus erythematosus disease activity index-2000.

## Data Availability

The data sets supporting the conclusions of this article and its supporting information are available from the corresponding author upon reasonable request.
